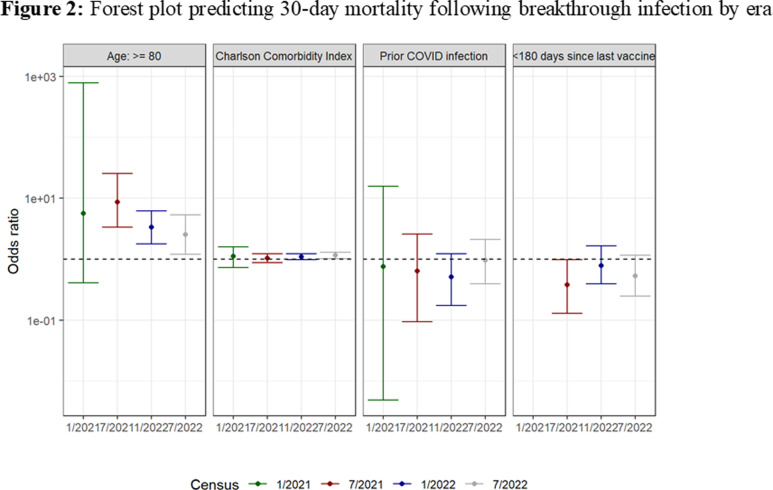# 34 Factors associated with hand hygiene and personal protective equipment compliance across a 9-hospital system in North Carolina

**DOI:** 10.1017/ash.2026.10476

**Published:** 2026-06-23

**Authors:** Brigid Wilson, Taissa Bej, Corinne Kowal, Federico Perez, Robin Jump

**Affiliations:** 1 Northeast Ohio VA Healthcare System; 2 VA Northeast Ohio Healthcare System; 3 Department of Veteran Affairs; 4 VA Pittsburgh Healthcare System

## Abstract

**Background:** Nursing homes residents are at high risk of severe COVID-19 infections. Age-related immunosenescence blunts the response to routine vaccinations, such that the estimated vaccine effectiveness for COVID-19 is <33% in nursing home residents. We assessed risk factors associated with breakthrough COVID-19 infections (BT) among previously vaccinated residents of Veterans Affairs (VA) nursing homes, termed Community Living Centers (CLCs). Methods We characterized residents who had received the primary COVID vaccine series and were present in VA CLCs at four census dates: 1/1/21, 7/1/21, 1/1/22, and 7/1/22 which included the Delta (mid-2021), Omicron BA.1 (early 2022) and BA.4/BA.5 (mid-2022) peaks. In each of these census cohorts, we assessed COVID-19 booster vaccines and identified laboratory confirmed COVID cases. We identified time-at-risk for BT starting at 14 days post-primary series vaccination or the beginning of the 6-month interval; and extending until death, BT, 7 days after discharge, or the end of the 6-month interval. For each census date, we characterized risk factors for BT and death following BT using survival and logistic regression models, respectively. Results We identified Table 1). Cases of BT infection increased from 41 pre-Delta to <800 during the Omicron BA.1 and BA.4/BA.5 waves. Survival models indicated an increased risk of BT among residents aged ≥Figure 1). We did not detect significant effects of sex, race and ethnicity, or Charlson Comorbidity Index (CCI). Compared to the primary series alone, a single booster dose had a protective effect against BT infection during the July 2021 and January 2022 eras; we did not detect effects of additional boosters. Mortality models from all census dates included 2011 residents with BT. Age ≥Figure 2). Discussion Among vaccinated nursing home residents, age ≥

**Figure:**
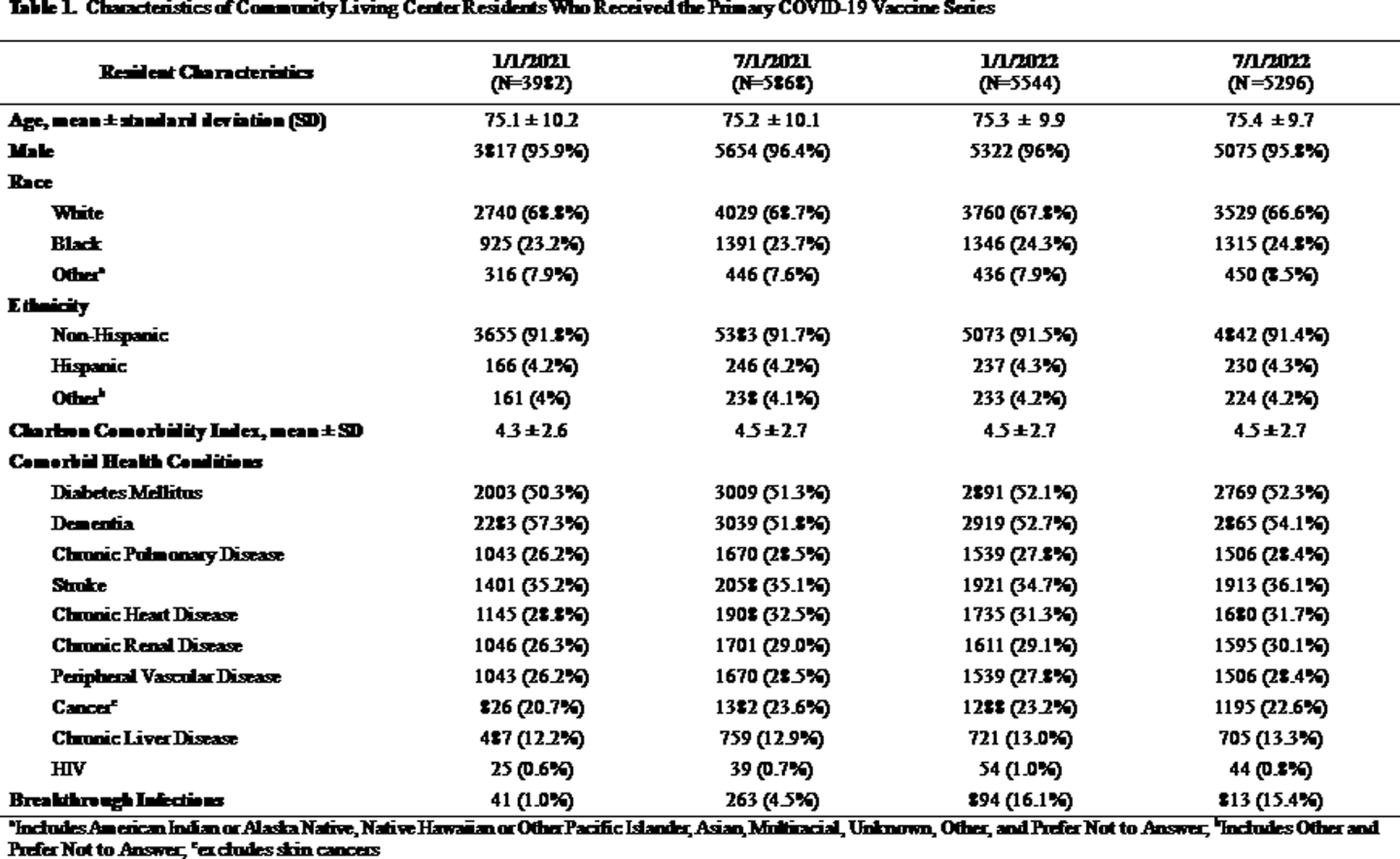

**Figure:**
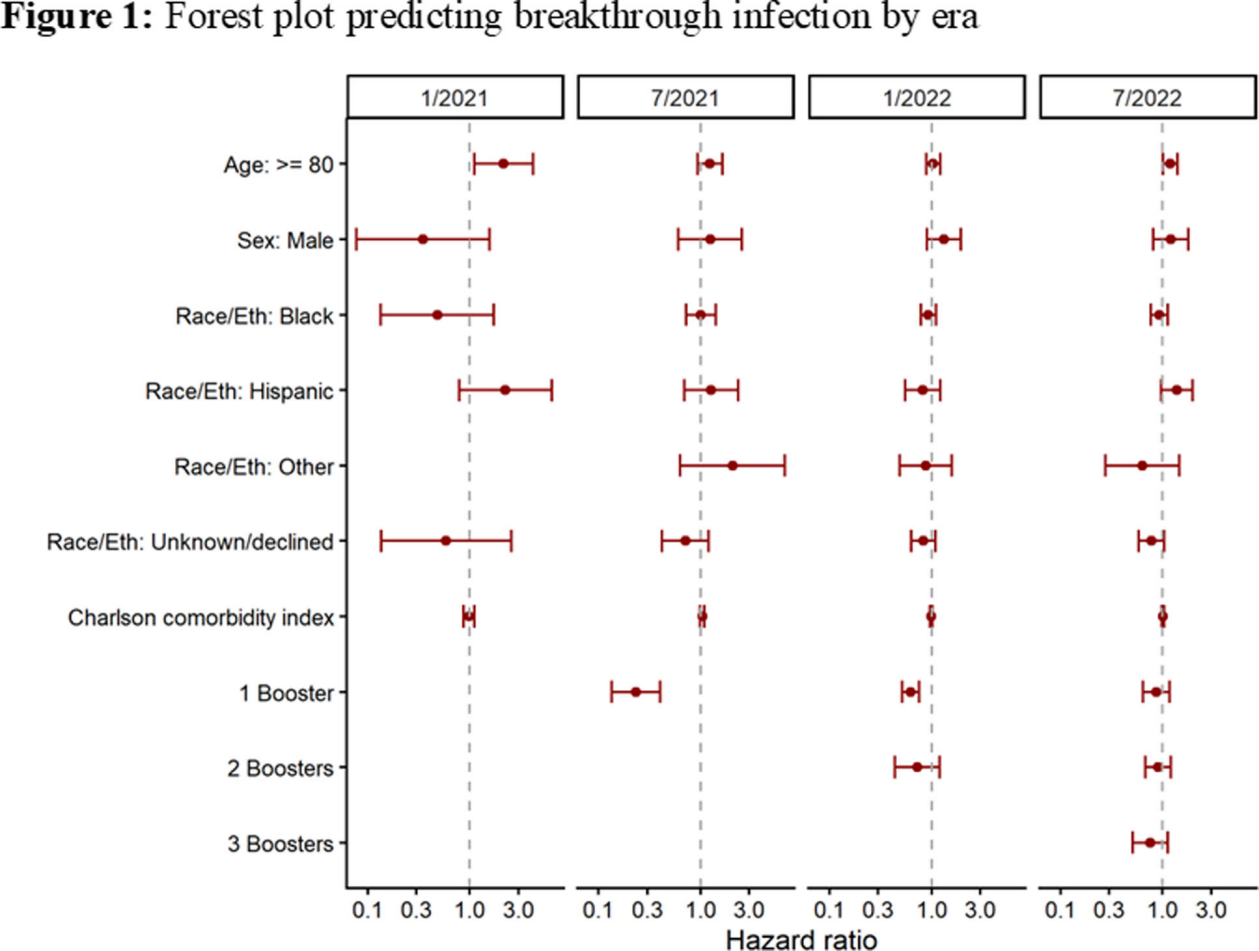

**Figure:**